# Retraction: Chaperonin CCT-Mediated AIB1 Folding Promotes the Growth of ERα-Positive Breast Cancer Cells on Hard Substrates

**DOI:** 10.1371/journal.pone.0202617

**Published:** 2018-08-15

**Authors:** The PLOS ONE Editors

After this article [1] was published, concerns were raised about similarities between the TCP-1 ε blot shown in Figure 4A and the CCTη blot for the Input samples in Figure 5A. The corresponding author, Dr. Luo, confirmed there was an error in image processing and requested retraction. The original data underlying the figure panels in question and other figures in the published article are no longer available.

In light of the concerns about Figures 4A and 5A and the unavailability of the underlying data, the *PLOS ONE* Editors retract this article.

The authors did not comment on the retraction decision.

## References

[1] Chen L, Zhang Z, Qiu J, Zhang L, Luo X, Jang J (2014) Chaperonin CCT-Mediated AIB1 Folding Promotes the Growth of ERα-Positive Breast Cancer Cells on Hard Substrates. PLoS ONE 9(5): e96085 10.1371/journal.pone.0096085 24788909 PMC4006900

